# Gingival recession and attachment loss: Cross-sectional and retrospective data of 10 years

**DOI:** 10.34172/japid.2021.007

**Published:** 2021-06-09

**Authors:** Ilma Robo, Saimir Heta, Geriona Lasku, Vera Ostreni

**Affiliations:** ^1^Department of Dentistry, Faculty of Medical Sciences, Albanian University, Albania; ^2^Department of Pediatric Surgery, QSUT, Albania

**Keywords:** Attachment loss, Cross-Sectional, Recession, Retrograde

## Abstract

**Background:**

Gingival recession is a manifestation of the presence of periodontitis and the expression of its characteristics for a long time in the patient’s oral cavity. Loss of attachment and its association with gingival recession affect the prosthetic value of the tooth as they significantly change the center of axial rotation of the tooth. The present study aimed to determine the correlation between gingival recession and attachment loss.

**Methods:**

Data on gingival recession and loss of attachment were collected in two groups of patients. In the first group (n=34), cross-sectional data were collected; in the second group (n=64), previously collected data over 10 years were evaluated.

**Results:**

Gingival recession was the most prevalent in the age group of 20-30 age group in 56% of the patients. The same values held for the retrograde data. An attachment loss of 4-6 mm was reported in 26% of the patients in the 31-50 age group in the cross-sectional data group, and 7 mm of gingival recession was reported in 3% of the patients in the 31-50 age group.

**Conclusion:**

The high prevalence of periodontitis at a young age indicates a poor prognosis of this disease at older ages. Gingival recession associated with attachment loss for patients with chronic periodontitis has higher values at the 31-50 age group, where systemic conditions are gradually developing in the human body.

## Introduction

The patient with periodontitis has *Actinomyces Actinomycetemcomitans,* a prepotent bacteria species against other members of the oral flora. It is the cause of the destruction of periodontal ligament fibers as it stimulates the release of prostaglandin E2 with a tendency to resorb collagen type III in periodontal ligament fibers.^1,2,3^ This bacterium is present with the corresponding protective immunoglobulins in the patient’s blood. The presence of this bacterium increases the tendency for rapid alveolar bone loss and horizontal bone loss during the stages when the patient passes from the inactive phase to the active phase of the disease. Horizontal bone loss exposes the tooth roots, changing the ratio of the clinical crown to the anatomic crown and the clinical root to the anatomic root. In the active stages of the disease, the generalized horizontal loss in patients with chronic periodontitis causes the exposure of root in the cervical area of affected tooth.^4,5^ As long as periodontitis is manifested with marked bone loss, exposed teeth increase vestibulo-lingual or palatal mobility.^2,6,7,8^

When the center of rotation of the tooth shifts further in the radicular direction, the prosthetic value of these teeth is reduced. The severity of the chronic periodontal disease is directly proportional to the value of bone resorption, mainly of the horizontal type in the affected teeth, and inversely proportional to the prosthetic value of the tooth. The latter is explained by the movement in the apical direction of the tooth center of rotation along the horizontal axis.^1,2,5,8,9^ The most common transition is vertical, from mother to child, and even from one family member to another. This last possibility depends on the family customs.^10^

Periodontitis is not associated with systemic symptoms, body temperature, or swelling of the local lymph nodes. Chronic periodontitis is an infection that expresses its activity locally with pronounced bone resorption in the affected teeth. There is usually a generalized appearance in the complete teeth in the oral cavity of the affected patient and not in a set of teeth. Patients with chronic periodontitis are prone to pronounced atherosclerosis. If they are female patients, they are prone to premature birth. These are the two relationships of periodontal medicine with periodontitis, where the role of the dentist lies in warning against what might happen to prevent the effect of the bacterium responsible for chronic periodontitis, i.e., *A. Actinomycetemcomitans*.^4,5,11,12^

The collection and processing of data during the application of retrospective studies requires human resources and very specific tools to achieve the study’s aims during long-term studies. The aim of a retrospective study is something that can be decided based on the specificity of the diagnostic tool and the number of human resources on which data collection and processing is based. In chronic periodontitis, we are talking about periodontal indices that mainly depend on figures to show the level of bone loss at specific tooth surfaces. For example, the bleeding index is quite sensitive when bleeding is assessed, like the spontaneous one after careful periodontal probing. The same can be said for cross-sectional studies, where data processing is applied to a large number of patients or subjects. Cross-sectional studies performed on a large number of subjects require conscious human resources for the application of specific periodontal diagnostic tools.^1,4,13,14^

## Methods

Analyzing cross-sectional and retrospective data on gingival recession and attachment loss due to periodontitis in the active or inactive phases would require coping with figures recorded and conceived at two different times. The purpose of the study was to record data on recession and attachment loss at the most diverse and varied age intervals. Therefore, the data were collected in two groups of patients. The first group included patients presenting ad hoc at the Dental Clinic of Albanian University from October 2019 to January 2020 for periodontal treatment. The second group of patients consisted of the same patients of the Dental Clinic of Albanian University but presented in October-November in the annual interval 2010-2018 (every year in the mentioned monthly period).

The patients of the first group agreed to be included in the study verbally while maintaining the anonymity of the recorded data. For the patients of the second group, the data recording was performed in the completed periodontal cards at the respective times. Each periodontal card was signed by the patient.

Attachment loss and sulcus depth are quite popular considerations in periodontology. These notions might have the same values, as the distance from the cementoenamel junction (CEJ) to the pocket depth may be the same as the distance from the gingival margin to the pocket depth. Normally, this picture does not match the characteristics of chronic periodontitis. Attachment loss might be less than the distance in millimeters from the gingival margin to the depth of the sulcus. This is the typical clinical picture of gingival hypertrophy regardless of the cause of its onset. One of the most common causes is inflammation, which presents with inflammatory gingival hypertrophy.^15^

The clinical picture consistent with the presence of periodontitis is precisely when the attachment loss is greater than the depth of the sulcus, i.e., the gingival margin exposes the root surface to the oral cavity, increasing the ratio of the clinical crown to the clinical root. These data were recorded for the patients included in the study in both groups. The 3-mm value is the limit for both notions to separate the physiological status from the pathological condition. A 3-mm gingival sulcus depth is a normal or physiological norm. In the loss of attachment, when the sulcus depth and attachment loss are 3 mm, we can say that we are within the physiological norm. A pathological condition is considered when either the attachment loss or the sulcus depth is >3 mm.^16-19^

The 3-mm value in periodontology is also called the limit value between the ability to treat patients with periodontal therapy or with periodontal surgery. The Williams probe was used to measure sulcus depth or attachment loss. This periodontal probe is easily applicable for routine measurements in periodontology.

## Results

**Figure 1:**
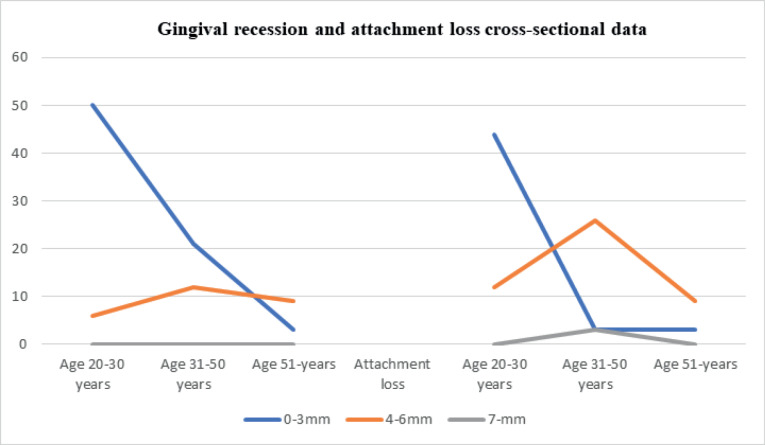
This graph presents the data on gingival recession and attachment loss for the first group of patients in terms of age (cross-sectional).

**Figure 2:**
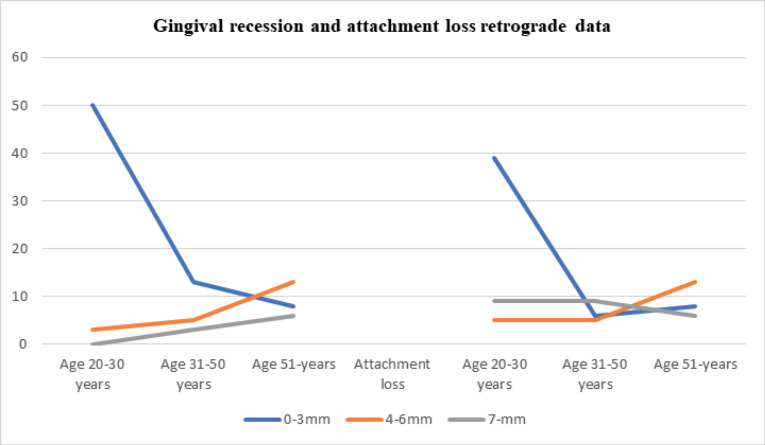
This graph presents the data on gingival recession and attachment loss for the second group of patients in terms of age (retrograde).

**Figure 3:**
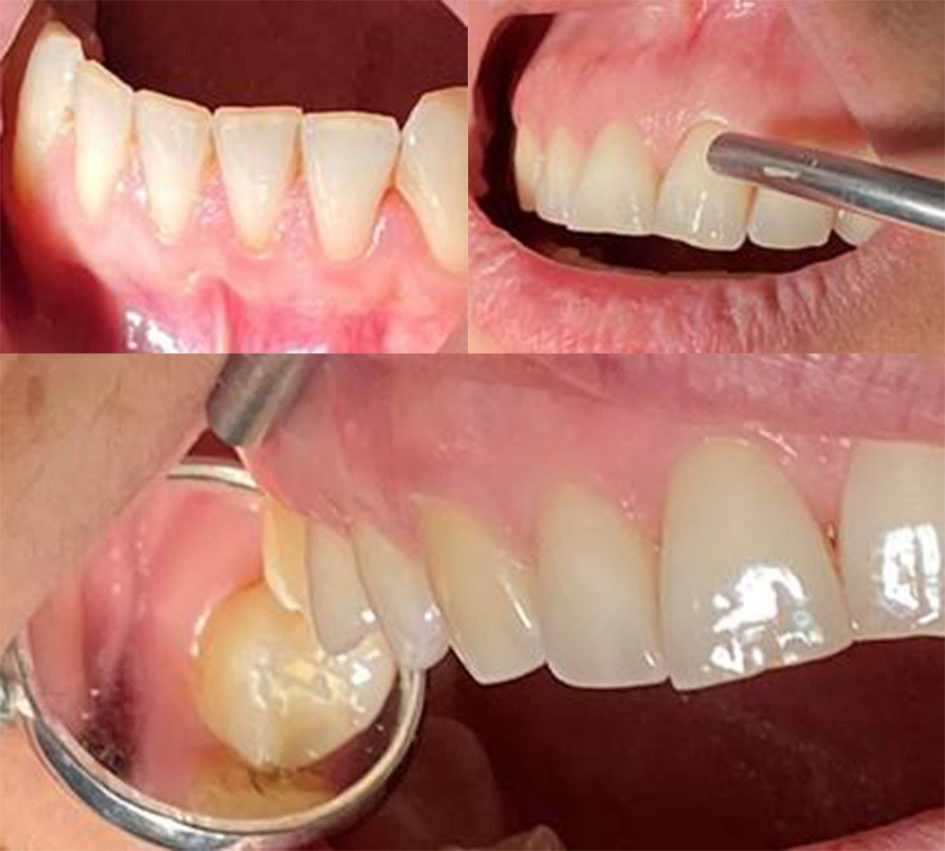
Patients with periodontitis, age 33 years, where there is an almost total absence of bacterial plaque, even in the maxillary molars. The pink color of the gingiva and orange peel is a clinical sign of gingival health.

**Figure 4:**
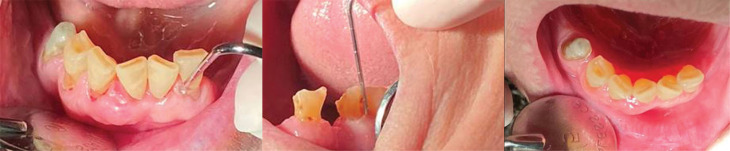
Photo of a 92-year-old patient in the group of patients for cross-sectional data, showing the evaluation of the bleeding index after probing and measuring attachment loss. We also draw attention to the presence of pronounced gingival recessions.

The data collected according to the two specific groups of patients included in the study are presented in Tables 1-4 and Figures 1-4

**Table 1:** Cross-sectional data on gingival recession for the first group of patients, classified according to patient age

**Gingival recession**	**0-3 mm**	**%**	**4-6 mm**	**%**	**7 mm**	**%**	**Total**	**%**
Patients								
20-30 age group	17	50%	2	6%	0	0%	19	56%
31-50 age group	7	21%	4	12%	0	0%	11	33%
>51 age group	1	3%	3	9%	0	0%	4	12%
Total	25	74%	9	26%	0	0%	34	100%

**Table 2:** Retrograde data for gingival recession for the second group of patients, classified by age

**Gingival recession**	**0-3 mm**	**%**	**4-6 mm**	**%**	**7 mm**	**%**	**Total**	**%**
Patients								
20-30 age group	32	50%	2	3%	0	0%	34	56%
31-50 age group	8	13%	3	5%	2	3%	13	33%
>51 age group	5	8%	8	13%	4	6%	17	12%
Total	45	70%	13	21%	6	9%	64	100%

**Table 3:** Attachment loss data for the first group of patients, by age

**Attachment loss**	**0-3 mm**	**%**	**4-6 mm**	**%**	**7 mm**	**%**	**Total**	**%**
Patients								
20-30 age group	15	44%	4	12%	0	0%	19	56%
31-50 age group	1	3%	9	26%	1	3%	11	33%
>51 age group	1	3%	3	9%	0	0%	4	12%
Total	17	50%	16	47%	1	3%	34	100%

**Table 4:** Attachment loss data for the second group of patients, by age

**Attachment loss**	**0-3 mm**	**%**	**4-6 mm**	**%**	**7 mm**	**%**	**Total**	**%**
Patients								
20-30 age group	25	39%	3	5%	6	9%	34	56%
31-50 age group	4	6%	3	5%	6	9%	13	33%
>51 age group	5	8%	8	13%	4	6%	17	12%
Total	34	53%	14	23%	16	24%	64	100%

## Discussions

Gingival recession was most prevalent in the 20-30 age group in up to 56% of patients. The same values ​​were recorded in retrograde data. In cross-sectional data, there was a gingival recession of 0-3 mm in about 50% of cases in the 20-30 age group, with 21% of cases in the 31-50 age group. A gingival recession of 4-6 mm was recorded in the 31-50 age group in 12% of the cases included in the study. Gingival recessions ​​of >7 mm were not found in patients included in the cross-sectional group. For retrograde data on gingival recession, there was 4-6 mm of gingival recession in about 13% of the subjects >51 years of age, with 7 mm of gingival recession in 3% of the patients in the 31-50 age group and 6% of the patients >51 years of age. An attachment loss ​​of 4-6 mm was recorded in 26% of the subjects in the 31-50 age group in the cross-sectional data group, with 7 mm in 3% of the subjects in the 31-50 age group.

For the retrograde data set, about attachment loss it’s documented that 13% of patients aged 51 years and older, showed attachment losses in values ​​of 4-6mm. 9% of patients of age 31-50 years old are presented with attachment loss of value 7-mm. 9% of patients of age 20-30 years old are presented also with 7mm value of attachment loss.

Chronic periodontitis has various risk factors, including smoking. Various studies in the literature have compared the effect of this risk factor in the smoking group with the non-smoking control group.^2,5,14,17,21,22^ The earliest study was conducted in the US in 1971-1974 to assess the prevalence of periodontal diseases in general in a relatively large sample, i.e., 28,000 patients. In this study, the prevalence of aggressive periodontitis was about 10%. We quote: “Patients with the localized form (5.23%) were slightly higher compared to those with the generalized form (4.45%).” The results are consistent with those reported by Sharma and Rai,^23^ saying that those diagnosed with LAP (57%) were slightly higher compared to those diagnosed with GAP (43%). In contrast, in a study by Imran and on Yemeni students, individuals with LAP (2.6%) were much more numerous compared to those with GAP (1%). Similarly, Kumar et al^24^ describe a higher prevalence of LAP (71%) compared to GAP (29%). The change in the prevalence ratios in these previous studies compared to the current study might reflect the influence of genetic and environmental factors due to different geographical locations.^1,9,11,12,19^

In another study involving 7178 patients with aggressive periodontitis, 3537 were male, and 3641 were female, with a corresponding ratio 0.9, indicating a positive relationship between females and aggressive periodontitis.^18^ These results are similar to those reported by Cortelli et al,^19^ and Hormand, Frandsen, and Baer.^20^ In the current study, 67,236 patients were diagnosed with chronic periodontitis, which was significantly higher than that of aggressive periodontitis, which accounted for only 7,178 patients. This is consistent with the results reported by Sharma and Rai.^23^ The results of the study helped determine the different forms of periodontitis in the population of Hyderabad.^3,7,12,21^ This might serve as a basis for future studies to find ways to improve oral health in the population.

The prevalence of moderate to severe chronic periodontitis was 74%. Coronary artery disease is associated with the severity of periodontal disease (P=0.02). Periodontal treatment reduced the 24-month incidence of cardiovascular events and cardiovascular death, suggesting that periodontal treatment might improve cardiovascular outcomes. We also suggest periodontal treatment in patients with kidney disease. Assess the condition after periodontal therapy in private practice and identify the risk factors that lead to the recurrence of the disease and tooth loss. One hundred patients who participated in a routine visit were included in the study; all were treated for periodontal disease and were in maintenance (supportive therapy) for almost two years. 91% of participants had an initial diagnosis of aggressive, with 9% of chronic periodontitis. The mean participant age was 46, with 26 teeth; 283 of the 2,549 teeth present were initially extracted, half of which were molars. Periodontal and endo-periodontal complications were determined, with only 16 missing teeth. The prevalence of all the categories of probing depth (PD) decreased significantly.^2,5,8,21,22^

Tooth loss and tissue damage due to periodontal disease can be reversed if it is treated and if patients show proper care. In this clinical trial, eight patients with moderate chronic periodontitis and two-walled intraosseous defects were selected. Defects were assigned to four groups: debridement, 1% metformin, PRGF, and PRGF and metformin. The parameters of vertical probing depth, vertical attachment level, and gingival index were measured at baseline, immediately before surgery, and three and six months after surgery. Furthermore, radiographic changes were evaluated with digital radiographs before and six months after surgery. The analysis of the results was performed with repeated measurements by the Friedman test.^1,3,13,22^ All the groups showed improvements in all the clinical parameters after six months. Inter-group comparisons of GI, CAL, and PPD parameters did not reveal any statistically significant differences. Radiographic changes in the 1% metformin group with PRGF revealed statistically significant differences compared to the other groups. However, there were no statistically significant differences in the other groups.

This study aimed to determine the prevalence of chronic periodontitis (CP) in patients with chronic kidney disease (CKD) and ascertain its association with some indicator factors of micro-inflammation. A total of 135 patients with CKD in dialysis treatment were included in this study. Biochemical parameters, clinical binding level, and pocket depth were recorded according to the American Academy of Periodontology and the CDC (CDC-AAP). Gingivitis and CP were recorded based on the classification of periodontal biofilm-gingival disease (BGI). The non-response rate to the survey was 10%. A total of 2,636 teeth were examined in 135 patients, of whom 52.5% were male. The mean age was 55.7 years; 41.4% had a history (history of smoking; 78 patients were on hemodialysis and 57 on peritoneal dialysis; 55.5% had been on dialysis for more than three years.^1,4,14,24^ The prevalence of gingivitis and periodontitis was 14.8% at 95% CI (9.7‒21.9) and 82.2% at 95% CI (74.7‒87.8), respectively, according to the BGI index. The severity of CP was: no periodontitis, 14.0% at 95% CI (9.1‒21.1); mild, 11.1% at 95% CI (6.7‒17.7); moderate, 28.8% at 95% CI (21.7‒37.1); and severe, 45.9% at 95% CI (31.6‒54.47). Peritoneal dialysis and time on dialysis >3 years increase the chance of developing periodontitis, OR=11.0 at 95% CI (2.2‒53.8) and OS=7.6 at 95% CI (1.1‒50.2), respectively. Given the high prevalence of CP in this population, programs should be established to provide better periodontal and gingival care in the dialysis population.^21,22^

Periodontitis is a chronic inflammation that destroys periodontal tissues caused by the accumulation of bacterial biofilms affected by environmental factors. This report describes an association study to assess the association of environmental factors with periodontitis using the National Health and Nutrition Survey (NHANES) from 1999 to 2004. A wide range of environmental variables (156) was assessed in patients categorized for periodontitis (n=8884). Numerous statistical approaches were used to explore this data and identify variable environmental patterns that increased or decreased the prevalence of periodontitis. Our findings showed that a set of environmental variables were different in periodontitis in smokers, ex-smokers, or non-smokers. Environmental factors included blood lead levels, selected nutrients, and PCBs. These factors were associated with more classical risk factors (e.g., age, gender, race/ethnicity) to create a pattern showing an increased prevalence of the disease up to 2-4 times throughout the population; environmental factors are statistically associated with the disease prevalence of periodontitis. Existing evidence suggests that these might contribute to altered gene expression and biological processes that enhance the destruction of inflammatory tissues.^14,18,19^ This study aimed to determine the prevalence of periodontitis among patients affected by various lung diseases in Moradabad district, Uttar Pradesh, India. A total of 700 patients suffering from lung disease, including tuberculosis (TB), chronic obstructive pulmonary disease, or pneumonia in the 12-70 age group were selected for the study. The periodontal disease index and the periodontal index for infection risk were recorded for all patients. The results were calculated and subjected to statistical analyses. The majority of the study population were diagnosed with periodontitis with a higher proportion in the highest risk category according to PIRI scores.^1,4,13,15,17^

## Conclusions

Gingival recession and attachment loss showed declining values when we compared cross-sectional and retrograde data on these concepts collected in patients included in this study. Patients’ sensitivity increases to the consequences of the existing periodontium in the oral cavity, which passes into active and invasive stages but does not fully heal. Patients’ access to and care for periodontal disease is on the increase.

## Author’s contributions

IR collected the scientific data and wrote the manuscript. SH revised and edited the manuscript. Literature research was conducted by SH. GL and VO collected the scientific data. All authors read and approved the final manuscript.

## Availability of data

The data from the reported study are available upon request from the corresponding author.

## Ethics approval

As the authors of the article, we state no violation of the code of ethics during the study. Patients gave consent for the publication of their data, including their images in the paper.

## Competing interests

The authors declare no competing interests.
